# Correlation between the expression of DNMT1, and GSTP1 and APC, and the methylation status of GSTP1 and APC in association with their clinical significance in prostate cancer

**DOI:** 10.3892/mmr.2015.3402

**Published:** 2015-03-03

**Authors:** WEIJIE ZHANG, HONGLIANG JIAO, XUDONG ZHANG, RUIHUA ZHAO, FENG WANG, WEI HE, HONG ZONG, QINGXIA FAN, LIUXING WANG

**Affiliations:** 1 Departments of Oncology, The First Affiliated Hospital of Zhengzhou University, Zhengzhou, Henan 450052, P.R. China; 2 Departments of Neurosurgery, The First Affiliated Hospital of Zhengzhou University, Zhengzhou, Henan 450052, P.R. China

**Keywords:** prostate cancer, benign prostatic hyperplasia, DNA (cytosine-5)-methyltransferase 1, glutathione S-transferase-P1, adenomatous polyposis coli, methylation

## Abstract

The aim of the present study was to investigate the correlation between the expression of DNA (cytosine-5)-methyltransferase 1 (DNMT1), glutathione S-transferase-P1 (GSTP1) and adenomatous polyposis coli (APC), and the methylation status of GSTP1 and APC in prostate cancer (PCa) and benign prostatic hyperplasia (BPH), and to examine its clinical significance. Immunohistochemistry and reverse transcription-polymerase chain reaction (RT-PCR) was used to detect the expression of DNMT1, GSTP1 and APC in 56 samples of PCa tissue and 10 samples of BPH tissue. Methylation-specific-PCR was used to detect the methylation status of the CpG island promoters of GSTP1 and APC. The positive rate of expression of DNMT1 in poorly-differentiated PCa, moderately-differentiated PCa, well-differentiated PCa and BPH was 86.7%, 70.6%, 55.6% and 30.0%, respectively (P<0.05); for GSTP1, the positive rate was 13.3%, 29.4%, 44.4% and 90.0%, respectively (P<0.05); and for APC, the positive rate was 23.3%, 47.6%, 55.6% and 70.0%, respectively (P<0.05). The correlation coefficient for the association between the expression of DNMT1 and GSTP1 was −0.891 (P<0.05). Between the expression of DNMT1 and APC, the correlation coefficient was −0.721 (P<0.05). GSTP1 and APC were hypermethylated in the majority of PCa tissue samples. The positive rate of methylation of these genes in poorly-differentiated PCa was 83.3% and 73.3%, respectively. By contrast, hypomethylation (or demethylation) was observed in BPH samples, in which the positive rate of methylation was 10.0% and 20.0%, respectively (P<0.05). The increased expression of DNMT1, and the reduced expression of GSTP1 and APC have an important role in the development of PCa. Due to the high expression of DNMT1 mRNA, it is likely that the hypermethylation of CpG islands contributed to the silencing of GSTP1 and APC in PCa tissues.

## Introduction

Prostate cancer (PCa) is the commonest type of cancer in males, and which was expected to account for 26% (220,800) of cases of cancer in males in the United States in 2015. PCa was expected to account for 9% (27,540) of all cancer-related mortality in males and continues to be the second leading cause of cancer-associated mortality in this group (1).

Common risk factors for the development of cancer, including obesity and smoking, have only a weak association with PCa. In addition to age, ethnicity and family history, DNA methylation is an epigenetic event that exhibits an association with increased incidence of PCa (2,3). DNA methylation affects cell function by altering gene expression, and refers to the covalent addition of a methyl group, catalyzed by DNA methyltransferases (DNMTs), to the 5-carbon of cytosine in a CpG dinucleotide. The majority of CpG islands are unmethylated and associated with genes capable of active transcription in normal cells. By contrast, numerous CpG islands exhibit aberrant hypermethylation, and consequent gene inactivation, in cancer cells (4). A number of inactivated genes, including glutathione-S-transferase-P1 (GSTP1) and adenomatous polyposis coli (APC) encode proteins that act as tumor suppressors. Silencing of these genes is associated with tumor initiation, development and progression (5). Hypermethylation of these genes is associated with PCa (6).

Reliance on a single molecular marker may have limitations in the diagnosis of PCa. Therefore, it may be advantageous to develop multiple sensitive and specific molecular markers to be used simultaneously (7,8). The aim of the present study was to examine the methylation of GSTP1 and APC, in order to determine whether GSTP1 and APC methylation may be associated with PCa and, if so, whether they are clinically significant markers of disease. The potential use of hypermethylated genes as biomarkers to detect PCa are discussed in the present study.

## Materials and methods

### Human tissue samples

The present study was approved by the ethics committee of Zhengzhou University (Zhengzhou, China), and written informed consent was obtained from all of the patients. The clinical and pathological data was obtained for 56 PCa tissue samples, collected from the Urology department of the First Affiliated Hospital of Zhengzhou University (Zhengzhou, China) between January 2010 and December 2012. The samples consisted of 4 PCa radical prostatectomy tissue specimens, 19 sections of transurethral resection tissue and 33 biopsy specimens. The samples were obtained from patients aged between 50 and 93 years, with a median age of 77 years. According to the standard Gleason grading system for PCa, 9 samples were identified as well-differentiated adenocarcinoma, 17 samples were moderately-differentiated adenocarcinoma and 30 samples were poorly-differentiated adenocarcinoma. Based on the American Jewett-Whitmore-Prout system, 4 samples were stage A, 8 samples were stage B, 11 samples were stage C and 33 samples were stage D. A total of 10 further samples were identified as benign prostatic hyperplasia (BPH). Patients with BPH underwent open surgical resection and tissue specimens obtained from these individuals were used as controls. These patients were aged between 55 and 80 years, with a median age of 68 years. Patients had not been previously treated with radiotherapy, chemotherapy or other treatments for cancer.

### Immunohistochemistry

Tissue samples from the PCa and BPH specimens were investigated using immunohistochemistry. Deparaffinized sections were stained using hematoxylin and eosin (H&E). For immunostaining of paraffin-embedded sections, the slides were deparaffinized in xylene (Beijing Chemical Reagent Company, Beijing, China) and rehydrated in a graded alcohol series. Endogenous biotin was blocked using the SP-9000 kit (ZSGB-Bio, Beijing, China). Immunostaining was performed with three antibodies: Anti-DNMT1 mouse monoclonal antibody (cat. no. ab54759; Abcam, Cambridge, UK; 1:500); anti-GSTP1 mouse monoclonal antibody (cat. no. sc-376013; Santa Cruz Biotechnology, Inc., Santa Cruz, CA, USA; 1:500) and anti-APC rabbit polyclonal antibody (cat. no. sc-20987; Santa Cruz Biotechnology, Inc.; 1:500). Incubation with the primary antibodies was performed in a humidified chamber at 37°C for 1 h. The goat anti-rabbit immunoglobulin G secondary antibody (cat. no. SP9000; Beijing Zhongshan Golden Bridge Biotechnology Co., Ltd., Beijing, China; 1:5,000) was then added and incubated for 30 min at room temperature. The slides were washed between steps with Tris-buffered saline. Immunoreactions were visualized via a streptavidin-biotin complex, using a 3,3′-diaminobenzidine chromogenic kit (ZSGB-Bio). The specimens were subsequently counter-stained with hematoxylin. The omission of primary antibodies served as a negative control.

### RNA isolation and semi-quantitative reverse transcription-polymerase chain reaction (RT-PCR) analysis

Total RNA was extracted from PCa and BPH specimens using TRIzol reagent (Gibco-BRL, Gaithersburg, MD, USA). cDNA was synthesized from 1 *μ*g of RNA using the Thermoscript reverse transcriptase system (Fermentas, Burlington, ON, Canada). DNMT1 mRNA was amplified The following primer sequences were used: Forward 5′-CTA CCA GGG AGA AGG ACA GG-3′ and reverse: 5′-CTC ACA GAC GCC ACA TCG-3′ for DNMT1, forward: 5′-GCC CTA CAC CGT GGT CTA TT-3′ and reverse: 5′-GAC GCA GGA TGG TAT TGG A-3′ for GSTP1, forward: 5′-CCA ACA AGG CTA CGC TAT-3′ and reverse: 5′-TCT GCT CGC CAA GAC AAA-3′ for APC and forward: 5′-AGG CAT TGT GAT GGA CTC CG-3′ and reverse: 5′-AGT GAT GAC CTG GCC GTC AG-3′ for β-actin. β-actin was used as an internal control. The primer pair amplified a 152 base pair (bp) fragment for DNMT1, a 209 bp fragment for GSTP1, a 127 bp fragment for APC and a 301 bp fragment for β-actin. PCR was performed in a thermal cycler (GeneAmp PCR system 9700; Applied Biosystems, Foster City, CA, USA) for 35 cycles, consisting of denaturation at 94°C for 30 sec, annealing at 65°C for 45 sec (β-actin) or 54°C for 30 sec (DNMT1, GSTP1 and APC), and extension at 72°C for 90 sec, followed by a final 5 min extension at 94°C. The reaction products were loaded onto 1.5% agarose gels containing ethidium bromide and visualized under Biospectrum 600 imaging system (UVP, Upland, CA, USA). The band intensities of the PCR products were analyzed using the UVP VisionWorks LS 6.6a system (UVP) and are expressed as the mean ± standard deviation.

### Methylation-specific PCR (MS-PCR)

Genomic DNA samples from PCa and BPH tissue were isolated using a DNA extraction kit (Axygen Biotechnology, Hangzhou, China). Genomic DNA (1 *μ*g) was treated with sodium bisulfite using the CpGenome™ DNA modification kit (Epigentek, Brooklyn, NY, USA). MS-PCR was performed in the thermal cycler (GeneAmp PCR system 9700; Applied Biosystems). For GSTP1, the following primer sequences were used: Forward: 5′-GAT GTT TGG GGT GTA GTG GTT GTT-3′ and reverse: 5′-CCA CCC CAA TAC TAA ATC ACA ACA-3′ for unmethylated GSTP1 DNA and forward: 5′-TTC GGG GTG TAG CGG TCG TC-3′ and reverse: 5′-GCC CCA ATA CTA AAT CAC GAC G-3′ for methylated GSTP1 DNA. PCR amplification of the GSTP1 gene was conducted as follows: 94°C for 5 min, followed by denaturation at 94°C for 30 sec, annealing at 58°C for 45 sec and extension at 72°C for 90 sec, followed by a final 5 min extension at 94°C for 35 cycles. The primers amplified a 97(u)/93(m)-bp fragment, respectively. For APC, the following primers were used: Forward: 5′-GTG TTT TAT TGT GGA GTG TGG GTT-3′ and reverse: 5′-AAC CAA TCA ACA AAC TCC CAA CAA-3′ for unmethylated APC DNA and forward: 5′-TAT TGC GGA GTG CGG GTC-3′ and reverse: 5′-TCG ACG AAC TCC CGA CGA-3′ for methylated APC DNA. PCR amplification of the APC gene was conducted as follows: 94°C for 5 min, followed by denaturation at 94°°C for 30 sec, annealing at 59°C for 45 sec and extension at 72 for 90 sec, followed by a final 5 min extension at 94°C for 35 cycles. The primers amplified a 111(u)/98(m)-bp fragment, respectively. The reaction products were loaded onto 2% agarose gels containing ethidium bromide (Beijing Chemical Reagent Company) and visualized under Biospectrum 600 imaging system (UVP). The band intensities of the PCR products were analyzed using UVP VisionWorks LS 6.6a (UVP) and are expressed as the mean ± standard deviation.

### Statistical analysis

Data was analyzed statistically using the SPSS 17.0 package (SPSS, Inc., Chicago, IL, USA). The results are expressed as the mean ± standard deviation. The χ^2^ test and Spearman’s rank correlation coefficient analysis were used to assess the univariate association between the correlation of the expression of DNMT1, GSTP1 and APC, and the methylation status of GSTP1 and APC and the clinical significance of this marker. P<0.05 was considered to indicate a statistically significant difference.

## Results

### Immunohistochemical analysis of the expression of DNMT1, GSTP1 and APC in PCa and BPH tissues

**Figure 1:**
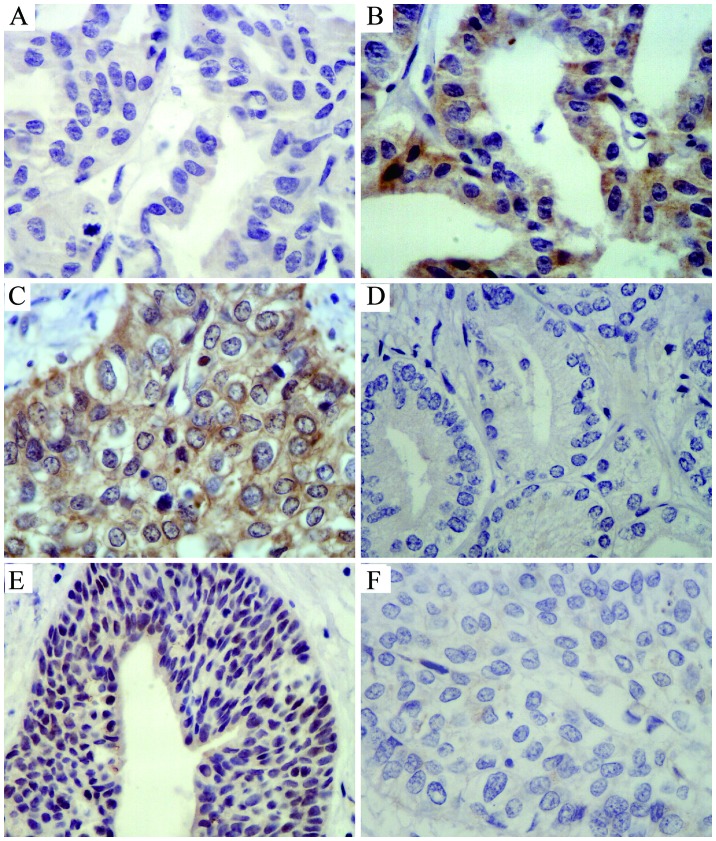
Immunohistochemical analysis of the expression of DNMT1, GSTP1 and APC proteins in PCa and benign prostatic hypertrophy (magnification, x400; Hoechst 33342 staining). (A) Negative expression of DNMT1 in PCa; (B) positive expression of DNMT1 in PCa; (C) positive expression of GSTP1 in PCa; (D) negative expression of GSTP1 in PCa; (E) positive expression of APC1 in PCa; and (F) negative expression of APC1 in PCa. DNMT1, DNA (cytosine-5)-methyltransferase 1; GSTP1, glutathione S-transferase-P1; APC, adenomatous polyposis coli; PCa, prostate cancer.

DNMT1 expression in the majority of cases of PCa was characterized histopathologically by yellow to brown nuclear staining, partial staining of the cytoplasm, with a focal or diffuse distribution to the staining. GSTP1 was expressed throughout the basal cell cytoplasm and exhibited partial nuclear staining in BPH tissues, with minimal staining in the nucleus and cytoplasm of cells from cancerous tissue. The APC protein was expressed predominantly in the cytoplasm in BPH tissues, and was negatively expressed in PCa tissues (Fig. 1, Table I).

### Association of the expression of DNMT1, GSTP1 and APC in PCa and BPH

DNMT1 expression and GSTP1, APC expression was negatively correlated in PCa and BPH (r_s_=−0.891, P<0.0001). Furthermore, DNMT1 expression and APC expression was negatively correlated in PCa and BPH (rs=−0.721, P<0.0001; data not shown).

### Expression of DNMT1, GSTP1 and APC mRNA in PCa and BPH

**Figure 2:**
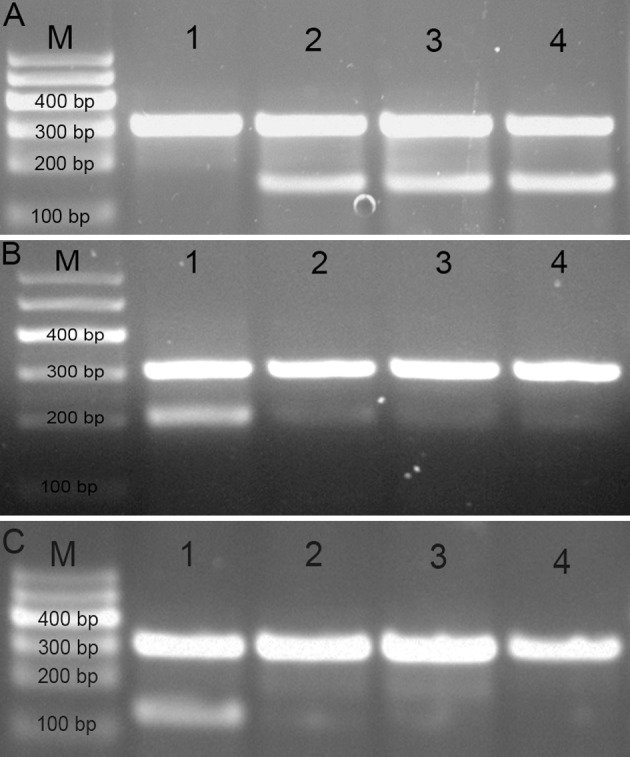
Expression of DNMT1, GSTP1 and APC mRNA in PCa and BPH. (A) mRNA expression of DNMT1 mRNA (152 bp); (B) GSTP1 mRNA (209 bp); and (C) APC mRNA (127 bp). The lanes are as follows: M, marker; 1, BPH; 2, well-differentiated PCa; 3, moderately- differentiated PCa; and 4, poorly-differentiated PCa. DNMT1, DNA (cytosine-5)-methyltransferase 1; GSTP1, glutathione S-transferase-P1; APC, adenomatous polyposis coli; PCa, prostate cancer; BPH, benign prostatic hyperplasia.

DNMT1 mRNA was highly expressed in the majority of PCa tissues (the positive rate in poorly-differentiated tissue was 90.0%), whereas a low level of expression was observed in BPH tissues (the positive rate was 40.0%). This difference was statistically significant (P<0.05). GSTP1 and APC mRNA was highly expressed in the majority of BPH tissues (the positive rates were 90.0% and 80.0%, respectively), while a low level of expression was observed in the majority of PCa tissues (the positive rate in poorly-differentiated tissues was 23.3% and 33.3%, respectively; P<0.05; Fig. 2 and Table II).

### Methylation status of the GSTP1 and APC genes in BPH and PCa tissues

**Figure 3:**
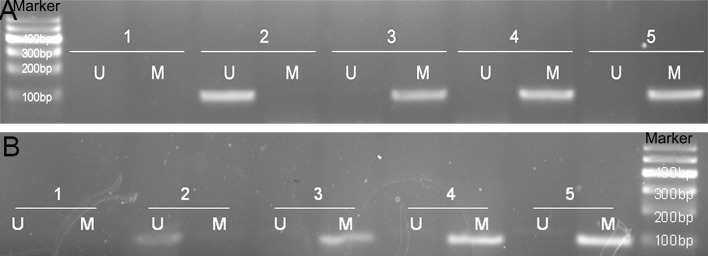
Methylation-specific polymerase chain reaction analysis of the methylation status of GSTP1 and APC in BPH and PCa. (A) methylation status of the GSTP1 (U: 97 bp; M: 93 bp); (B) APC (U: 111 bp; M: 98 bp). The lanes are as follows: Marker; 1, H_2_O; 2, BPH; 3, well-differentiated PCa; 4, moderately-differentiated PCa; and 5, poorly-differentiated PCa. U, unmethylated; M, methylated; BPH, benign prostatic hyperplasia; DNMT1, DNA (cytosine-5)-methyltransferase 1; GSTP1, glutathione S-transferase-P1; APC, adenomatous polyposis coli; PCa, prostate cancer.

The MS-PCR method was used to detect the methylation status of the GSTP1 and APC genes in 10 samples of BPH tissue and 56 samples of PCa tissue. GSTP1 and APC exhibited hypermethylation in the majority of the PCa samples (the positive rate in poorly-differentiated tissues was 83.3% and 73.3%, respectively), while hypomethylation (or demethylation) was observed in BPH samples (the positive rate was 10.0% and 20.0%, respectively, P<0.05; Fig. 3, Table III).

The association between the expression of DNMT1, and the methylation status of GSTP1 and APC in PCa tissue was assessed using Spearman’s rank correlation analysis. This demonstrated a significant positive correlation between DNMT1 expression and the methylation status of GSTP1 in PCa tissue (r_s_=0.817, P<0.0001). In addition, the correlation between DNMT1 expression and the methylation status of APC in PCa tissues was also significantly positive (r_s_= 0.671, P<0.0001)

## Discussion

Within the human genome, 70–80% of all CpG dinucleotides are methylated, and occur in repetitive and non-transcribed DNA regions (9). CpG dinucleotides also occur in transcribed DNA regions, which are clustered in CpG islands. CpG islands (typically 0.5–2 kb long) are located in the proximal promoter regions of numerous human genes (10). Cancer-dependent epigenetic regulation of genes involved in cell cycle control, DNA damage repair, tumor-cell metastasis/adhesion and hormonal responses, in addition to the silencing of these genes, is associated with tumor initiation, development and progression, thereby, increasing the risk of developing PCa (11).

The pattern of epigenetic change or altered gene transcription/expression is important in a number of types of cancer, including breast cancer and PCa (12). DNMTs transfer the methyl group from s-adenosylmethionine, thereby generating patterns of genomic methylation (13–15). To date, the following DNMTs have been identified: DNMT1, DNMT2, DNMT3a, DNMT3b and DNMT3L (16). DNMT1 is primarily responsible for the maintenance of DNA methylation. It has been observed in PCa, that there are numerous CpG islands exhibiting hypermethylation. In the present study, the expression of DNMT1 was examined in PCa and BPH using immunohistochemical methods. The expression of DNMT1 mRNA was lower in BPH than that in PCa tissues. In addition, the positive rate was significantly higher in poorly-differentiated PCa tissue, compared with that in BPH tissue, and this difference was statistically significant (P<0.05). The positive rate of GSTP1 and APC mRNA expression in BPH tissues was higher than that in poorly-differentiated PCa tissues; a difference that was also statistically significant (P<0.05). DNMT1 expression increased in PCa and this increased activity was associated with an increased degree of malignancy in the PCa cells.

GSTP1 acts to protect cells from DNA damage and the development of cancer, as it is associated with the detoxification, metabolism and elimination of potentially genotoxic exogenous compounds. Inhibition of the activity of GSTP1 may lead to increased DNA damage and susceptibility to cancer (17,18). The methylation of the GSTP1 gene promoter in PCa was first reported in 1994 (19) and is the most commonly detected epigenetic alteration, occurring in >90% of cancerous samples and ~70% of prostatic intraepithelial neoplasia (PIN) samples. However, it is rarely observed in BPH or normal prostate tissues (20). It has also been detected in proliferative inflammatory atrophy (PIA) lesions, although the mechanism by which GSTP1 subsequently alters methylation and silences transcription during progression from PIA to PIN remains to be elucidated (21). The expression of GSTP1 was examined in the BPH and PCa tissue samples using the same immunohistochemical method as a previous study by this group (22), in which it was demonstrated that with an increased level of methylation, the expression of GSTP1 is decreased, and a higher degree of malignancy is observed. It is hypothesized that GSTP1 promoter CpG island hypermethylation is one of the factors leading to its inactivation.

APC is an important tumor suppressor gene located on chromosome 5q21 (23). The APC protein performs a number of functions, including the regulation of β-catenin, which is an important component of the Wnt signaling pathway. APC regulates β-catenin stability by mediating β-catenin degradation. Loss of APC or β-catenin function leads to the activation of certain downstream target genes, including cyclin D1, c-myc and matrilysin (24). One possible mechanism for this is functional inactivation of the APC gene caused by promoter hypermethylation. In the present study, the expression of APC was examined in BPH and PCa tissue samples using an immunohistochemical method. It was shown that in PCa, compared with BPH, the APC methylation levels are increased and its expression is decreased. Thus, methylation may be associated with the loss of expression of APC and may have an important role in the development of PCa.

In conclusion, high expression of DNMT1 and low expression of GSTP1 and APC in PCa, indicates that promoter region hypermethylation of these genes is associated with tumor suppressor gene inactivation. GSTP1 and APC promoter CpG island hypermethylation, in particular that of GSTP1, may be used in the molecular diagnosis of PCa as an early marker of this disease (25). In addition, DNA methylation of does not alter the nucleotide sequence. Instead, it changes the level of chromosome structure and composition of protein acetylation, gene transcription indirectly causes inhibition (3). Thus, it is hypothesized that if a reduction in DNMT activity has the potential to restore the expression of certain tumor suppressor genes, an alteration in the methylation of this gene may be of use in the development of novel cancer therapies.

## Figures and Tables

**Table I:** Expression of DNMT1, GSTP1, APC protein in prostate cancer and benign prostatic hyperplasia, as determined by immunohistochemistry.

Tissue	n	DNMT1	GSTP1	APC
BPH	10	3 (30.0)	9 (90.0)	7 (70.0)
Well-differentiated PCa	9	5 (55.6)	4 (44.4)	5 (55.6)
Moderately-differentiated PCa	17	12 (70.6)	5 (29.4)	8 (47.6)
Poorly-differentiated PCa	30	26 (86.7)^a^	4 (13.3)^b^	7 (23.3)^c^

a χ^2^=12.410, P=0.006, compared with BPH tissue.

b χ^2^=20.468, P=0.0008, compared with BPH tissue.

c χ^2^=8.399, P=0.038, compared with BPH tissue. BPH, benign prostatic hyperplasia; DNMT1, DNA (cytosine-5)-methyltransferase 1; GSTP1, glutathione S-transferase-P1; APC, adenomatous polyposis coli; PCa, prostate cancer; n, number.

**Table II:** Expression of DNMT1, GSTP1, APC mRNA in prostate cancer and benign prostatic hyperplasia (%), as determined by semi-quantitative polymerase chain reaction.

Tissue	n	DNMT1 mRNA	GSTP1 mRNA	APC mRNA
Poorly-differentiated PCa	30	27 (90.0)	7 (23.3)	10 (33.3)
Moderately-differentiated PCa	17	14 (82.4)	7 (41.2)	9 (52.9)
Well-differentiated PCa	9	6 (66.7)	5 (55.6)	7 (77.8)
BPH	10	4 (40.0)^a^	9 (90.0)^a^	8 (80.0)^a^

a P<0.05, compared with poorly-differentiated PCa tissue. BPH, benign prostatic hyperplasia; DNMT1, DNA (cytosine-5)-methyltransferase 1; GSTP1, glutathione S-transferase-P1; APC, adenomatous polyposis coli; PCa, prostate cancer; n, number.

**Table III:** Methylation status of the GSTP1 and APC genes in BPH and PCa (%), as determined by methylation-specific polymerase chain reaction.

Tissue	n	mGSTP1 (+)	mAPC (+)
Poorly-differentiated PCa	30	25 (83.3)	22 (73.3)
Moderately-differentiated PCa	17	11 (64.7)	9 (52.9)
Well-differentiated PCa	9	5 (55.6)	3 (33.3)
BPH	10	1 (10.0)^a^	2 (20.0)^a^

a P<0.05, compared with poorly-differentiated PCa tissue. BPH, benign prostatic hyperplasia; DNMT1, DNA (cytosine-5)-methyltransferase 1; GSTP1, glutathione S-transferase-P1; APC, adenomatous polyposis coli; PCa, prostate cancer; n, number; mGSTP1, methylated GSTP1; mAPC, methylated APC.

## References

[1] Siegel RL, Miller KD, Jemal A (2015). Cancer statistics, 2015. CA Cancer J Clin.

[2] Rodriquez C, Calle EE, Miracle-McMahill HL, Tatham LM, Wingo PA, Thun MJ, Heath CW (1997). Family history and risk of fatal prostate cancer. Epidemiology.

[3] Neugut AI, Chen AC, Petrylak DP (2004). The skinny on obesity and prostate cancer prognosis. J Clin Oncol.

[4] Esteller M, Corn PG, Baylin SB, Herman JG (2001). A gene hypermethylation profile of human cancer. Cancer Res.

[5] Li LC, Okino ST, Dahiya R (2004). DNA methylation in prostate cancer. Biochim Biophys Acta.

[6] Richiardi L, Fiano V, Vizzini L, De Marco L, Delsedime L, Akre O, Tos AG, Merletti F (2009). Promoter methylation in APC, RUNX3, and GSTP1 and mortality in prostate cancer patients. J Clin Oncol.

[7] Kang GH, Lee S, Lee H, Hwang J, Aberrant KS (2004). CpG island hypermethylation of multiple genes in prostate cancer and prostatic intraepithelial. Neoplasia J Pathol.

[8] Phé V, Cussenot O, Rouprêt M (2010). Methylated genes as potential biomarkers in prostate cancer. BJU Int.

[9] Yoder JA, Walsh CP, Bestor TH (1997). Cytosine methylation and the ecology of intragenomic parasites. Trends Genet.

[10] Bird AP (1986). CpG-rich islands and the function of DNA methylation. Nature.

[11] Gardiner-Garden M, Frommer M (1987). CpG islands in vertebrate genomes. J Mol Biol.

[12] Baylin SB, Ohm JE (2006). Epigenetic gene silencing in cancer-a mechanism for early oncogenic pathway addiction?. Nat Rev Cancer.

[13] Ross SA (2003). Diet and DNA methylation interactions in cancer prevention. Ann NY Acad Sci.

[14] Brenner C, Fuks F (2006). DNA methyltransferases: facts, clue, mysteries. Curr Top Microbiol Immunol.

[15] Gopisetty G, Ramachandran K, Singal R (2006). DNA methylation and apoptosis. Mol Immunol.

[16] Singal R, Ginder GD (1999). DNA methylation. Blood.

[17] Berhane K, Widersten M, Engstrom A, Kozarich JW, Mannervik B (1994). Detoxication of base propenals and other alpha, betaunsaturated aldehyde products of radical reactions and lipid peroxidation by human glutathione transferases. Proc Natl Acad Sci USA.

[18] Nelson CP, Kidd LC, Sauvageot J, Isaacs WB, De Marzo AM, Groopman JD, Nelson WG, Kensler TW (2001). Protection against 2-hydroxyamino-1-methyl-6-phenylimidazo[4,5-b]pyridine cytotoxicity and DNA adduct formation in human prostate by glutathione S-transferase P1. Cancer Res.

[19] Lee WH, Morton RA, Epstein JI, Brooks JD, Campbell PA, Bova GS, Hsieh WS, Isaacs WB, Nelson WG (1994). Cytidine methylation of regulatory sequences near the pi-class glutathione S-transferase gene accompanies human prostatic carcinogenesis. Proc Natl Acad Sci USA.

[20] Brooks JD, Weinstein M, Lin X, Sun Y, Pin SS, Bova GS, Epstein JI, Isaacs WB, Nelson WG (1998). CG island methylation changes near the GSTP1 gene in prostatic intraepithelial neoplasia. Cancer Epidemiol Biomark Prev.

[21] Nakayama M, Bennett CJ, Hicks JL, Epstein JI, Platz EA, Nelson WG, De Marzo AM (2003). Hypermethylation of the human glutathione S-transferase-pi gene (GSTP1) CpG island is present in a subset of proliferative inflammatory atrophy lesions but not in normal or hyperplastic epithelium of the prostate: a detailed study using laser-capture microdissection. Am J Pathol.

[22] Lin X, Tascilar M, Lee WH (2001). GSTP1 CpG island hypermethylation is responsible for the absence of GSTP1 expression in human prostate cancer cells. Am J Pathol.

[23] Thliveris A, Albertsen H, Tuohy T, Carlson M, Groden J, Joslyn G, Gelbert L, Samowitz W, Spirio L, White R (1996). Long-range physical map and deletion characterization of the 1100-kb NotI restriction fragment harboring the APC gene. Genomics.

[24] Fearnhead NS, Britton MP, Bodmer WF (2001). The ABC of APC. Hum Mol Genet.

[25] Woodson K, O’Reilly KJ, Hanson JC (2008). The usefulness of the detection of GSTP1 methylation in urine as a biomarker in the diagnosis of prostate cancer. J Urol.

